# Dopamine presynaptically and heterogeneously modulates nucleus accumbens medium-spiny neuron GABA synapses *in vitro*

**DOI:** 10.1186/1471-2202-7-53

**Published:** 2006-06-30

**Authors:** Daron Geldwert, J Madison Norris, Igor G Feldman, Joshua J Schulman, Myra P Joyce, Stephen Rayport

**Affiliations:** 1 Department of Neuroscience, NYS Psychiatric Institute, 1051 Riverside Drive, Unit 62, New York, NY 10032, USA; 2 Department of Psychiatry, Columbia University, 1051 Riverside Drive, Unit 62, NewYork, NY 10032, USA; 3 Center for Neurobiology & Behavior, Columbia University, 1051 Riverside Drive, Unit 62, New York, NY 10032, USA

## Abstract

**Background:**

The striatal complex is the major target of dopamine action in the CNS. There, medium-spiny GABAergic neurons, which constitute about 95% of the neurons in the area, form a mutually inhibitory synaptic network that is modulated by dopamine. When put in culture, the neurons reestablish this network. In particular, they make autaptic connections that provide access to single, identified medium-spiny to medium-spiny neuron synaptic connections.

**Results:**

We examined medium-spiny neuron autaptic connections in postnatal cultures from the nucleus accumbens, the ventral part of the striatal complex. These connections were subject to presynaptic dopamine modulation. D1-like receptors mediated either inhibition or facilitation, while D2-like receptors predominantly mediated inhibition. Many connections showed both D1 and D2 modulation, consistent with a significant functional colocalization of D1 and D2-like receptors at presynaptic sites. These same connections were subject to GABA_A_, GABA_B_, norepinephrine and serotonin modulation, revealing a multiplicity of modulatory autoreceptors and heteroreceptors on individual varicosities. In some instances, autaptic connections had two components that were differentially modulated by dopamine agonists, suggesting that dopamine receptors could be distributed heterogeneously on the presynaptic varicosities making up a single synaptic (*i.e*. autaptic) connection.

**Conclusion:**

Differential trafficking of dopamine receptors to different presynaptic varicosities could explain the many controversial studies reporting widely varying degrees of dopamine receptor colocalization in medium-spiny neurons, as well as more generally the diversity of dopamine actions in target areas. Longer-term changes in the modulatory actions of dopamine in the striatal complex could be due to plasticity in the presynaptic distribution of dopamine receptors on medium-spiny neuron varicosities.

## Background

The synaptic actions of dopamine (DA) in the striatal complex, the principal target of DA neurons in the CNS, may best be described as heterogeneous. In the striatal complex, DA neurons synapse on and in proximity to medium-spiny GABA neurons [1]. Medium-spiny neurons (MSNs) constitute 95% of the neurons in the area [2,3], receive feed-forward GABAergic inhibition from fast spiking interneurons [4], and extensive excitatory input from cortex and thalamus, and in the case of MSNs in the ventral striatal complex, or nucleus accumbens (nAcc), also from the amygdala and hippocampus [5]. MSNs give rise to a profusion of local axon collaterals that ramify within the dendritic fields of the parent cell [6], as well as to all the efferent projections from the striatal complex [7,8]. Since evoked GABAergic inputs are dominated by the small population of fast-spiking interneurons [4,9,10], discerning the function of MSN-to-MSN synapses proved challenging [11]. Recently, dual intracellular recordings in striatal slices [12] and paired whole-cell recordings both in explant cultures [13,14] and slices [13,15-18] have shown that MSN-to-MSN synapses function. Although the synapses appear to be weak, in numbers they account for two thirds of the GABAergic inputs to a given MSN neuron [9,16], so on a network level their function is likely to be quite significant [11,16]. The argument can be made that these synapses are a major target of DA action. Indeed, they show potent DA modulation via presynaptic D1-like receptors mediating facilitation and D2-like receptors mediating inhibition [9].

The cellular and subcellular distribution of DA receptors on individual MSNs – a critical determinant of DA action – has been the subject of considerable debate [19]. Part of the controversy devolves to making the distinction between the molecular biology and the pharmacology. Pharmacologically identified D1-like receptors comprise D1 and D5 receptors; D2-like receptors comprise D2, D3 and D4 receptors [20]. Initial *in situ *hybridization studies reported the segregation of D1 and D2 receptors to different populations of MSNs [21,22], and this was amply confirmed in single cell RT-PCR studies [23], and in expression patterns revealed in D1 and D2 BAC transgenics [24]. D1 receptor expressing MSNs contain SubstanceP and project to the ventral midbrain (the direct pathway) where they show robust D1-mediated presynaptic facilitation [25,26]. D2 receptor expressing MSNs contain enkephalin and project to the ventral pallidum (the indirect pathway) where they show D2-mediated presynaptic inhibition [27,28].

While single-cell RT-PCR studies confirmed the lack of D1/D2 receptor colocalization in MSNs, they also showed about a 50% colocalization of D1-like and D2-like receptors [23]. This was confirmed in double-label fluoroprobe studies of MSNs in culture [29], where receptors were identified pharmacologically. Other studies stand in further counterpoint to these results. In acutely dissociated striatal neurons, biochemical measures of DA modulation indicated near complete D1/D2 overlap [30]. Confocal immunocytochemical studies with D1 and D2 receptor-selective antisera reported near complete D1/D2 colocalization on MSNs identified by DARPP-32 expression [31]. While D1 and D2 receptors are differentially distributed on the two classes of MSNs, accounting for the segregation seen in the *in situ *studies, expression of the other DA receptors, together with lower levels of receptor expression that may be functionally significant, argue against a strict segregationist view at the functional, *i.e*. pharmacological level.

The strict segregationist view has been further confounded by single-cell, axon-tracing studies that have shown that there are no direct axonal projections from the striatum to the ventral midbrain. Rather, all MSNs project to the pallidum, with about a third terminating there, and the remaining two thirds going on to the entopeduncular nucleus and the ventral midbrain [32,33]. This anatomy is consistent with physiological studies, in which MSN-to-MSN connections were activated by antidromic activation from the ventral pallidum. This showed that Substance P-containing neurons are subject to D1 modulation at their projection synapses and D2 modulation at their local synapses (in the striatum), while enkephalin-containing neurons are subject to D2 modulation at their projection synapses and D1 modulation at their local synapses [9].

While the consensus is that there is about a 50% overlap in D1- and D2-like DA receptor expression at the cell body level, the projection synapses of MSNs show either D1 or D2 pharmacology. Surmeier *et al*. [19], in a prescient review, suggested the possibility that DA receptors might be trafficked differentially into the axons of individual MSNs, accounting for the differences seen between local DA actions reported in the striatum (both pre- and postsynaptic) and distal actions on MSN projection synapses. To examine DA modulation of individual MSN-to-MSN synapses, and to address the hypothesis that DA receptors show differential trafficking, we used postnatal cell culture to gain access to individual, identified MSN synapses. We have found that there is significant presynaptic DA modulation. Moreover it is heterogeneous, consistent with differential trafficking of DA receptors to different presynaptic varicosities in single neurons. Part of this work has appeared previously in abstract form [34-36].

## Results

### GABAergic connections of medium-spiny neurons in vitro

In postnatal cultures made from the nucleus accumbens, the ventral component of the striatal complex, 95% of the neurons are medium-sized, GABAergic [37], and bear dendritic spines [38], serving to identify them as the predominant MSN population. The GABA_A _antagonists Bicuculline (50 μM) or Gabazine (SR-95531, 10 μM) [39] invariably inhibited the synaptic actions of these neurons, confirming their identification as MSNs. We present data from 82 MSNs (of a total of 166 recorded), in which cells remained healthy throughout the experiment, with small, stable leak currents, and in which there was satisfactory drug perfusion (IPSCs inhibited to <5% with GABA_A _blockade at the beginning and end of experiments). Input resistances measured 503 ± 36 MΩ (mean ± s.e.m.).

**Figure 1:**
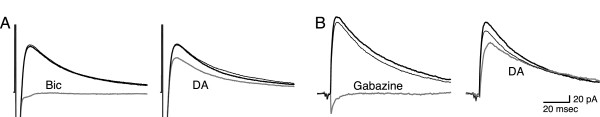
**Medium-spiny neuron autaptic and synaptic responses. A. Autaptic response inhibited by bicuculline**. Using a standard K-gluconate-based intracellular solution, an autaptic response (*left, thick black trace*) was evoked by a 1 msec depolarizing command, which triggered an inward action current (truncated off bottom of record, at start of trace). Local perfusion with the GABA_A _antagonist bicuculline (*gray trace*; Bic, 50 μM) completely blocked the response (leaving a small inward tail of the action current). The autaptic response recovered fully (*thin black trace*), nearly superimposing on the initial, control trace. Perfusion with dopamine (DA, 1 μM) inhibited the autaptic response (*right*), and this recovered fully. The wash trace in the left panel is the same as control trace in the right panel. Cells were stimulated throughout the experiment at 0.1 Hz to avoid frequency-dependent effects. Control, drug-treatment, and wash traces were collected after responses had stabilized, typically 30 sec after drug application or wash. Averages of 10 traces are shown. The amplitude of the initial control response was 140 pA, which was representative of the amplitude of the autaptic responses measured. **B.Synaptic current response inhibited by gabazine**. In another experiment, a nearby neuron was stimulated with a loose-patch electrode, evoking a synaptic response in the recorded MSN (*left, thick black trace*). Local perfusion with the GABA_A _antagonist gabazine (10 μM) completely (*gray trace*) blocked the response (leaving the small inward tail from the stimulus artifact). Following the wash (*thin black trace*), the synaptic response nearly recovered. Perfusion with DA (1 μM) inhibited the response (*right*), and this recovered with a second wash, although not completely. Traces shown are averages of 4 or 5 responses. Synaptic responses showed greater variation in amplitude than autaptic responses. The amplitude of the initial control synaptic response shown here was 220 pA, which was somewhat smaller than the typical synaptic response.

**Figure 2:**
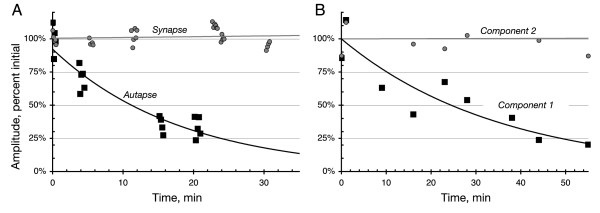
**Differential run down of autaptic *vs*. synaptic responses**. **A**. Control responses are presented from two experiments done on sister cultures recorded a day apart. While the synaptic response was stable for the duration of the experiment, the autaptic response showed significant run-down. **B**. In some recordings (see Figure 7) there was a second autaptic response (Component 2) riding on top of the initial response (Component 1), Component 1 ran down, while Component 2 did not, suggesting that Component 2 was mediated by autaptic connections made on more distal dendrites.

Stimulating cells with a brief depolarizing command (1 msec, 30 mV) evoked unclamped axon spikes that produced autaptic IPSCs with a rise time of 3.3 ± 0.40 msec and amplitude of 148 ± 23 nA (n = 15) (Figure 1A). Stimulation of neighboring medium-sized cell bodies with a loose patch electrode evoked IPSCs with a rise time of 1.2 ± 0.3 msec and amplitude 306 ± 85 nA (n = 9) (Figure 1B). Both the rise times (two-tailed t-test, p < 0.0005) and the amplitudes (p < 0.05) were significantly different. This would suggest that the faster, stronger synaptic inputs were more proximally distributed than the slower, weaker autaptic inputs. Autaptic IPSCs typically ran down over the course of 10–20 minutes and then stabilized at a plateau of reduced amplitude, while synaptic responses did not run down (Figure 2A). This would argue that washout affected mainly presynaptic mechanisms (as only autaptic responses were subject to wash-out). Nonetheless, we focused on autaptic responses, because they provided access to single identified MSN-to-MSN synapses. We never saw failures, even when responses had diminished, so the recorded IPSCs reflect the activation of multiple release sites.

### DA modulation

**Figure 3:**
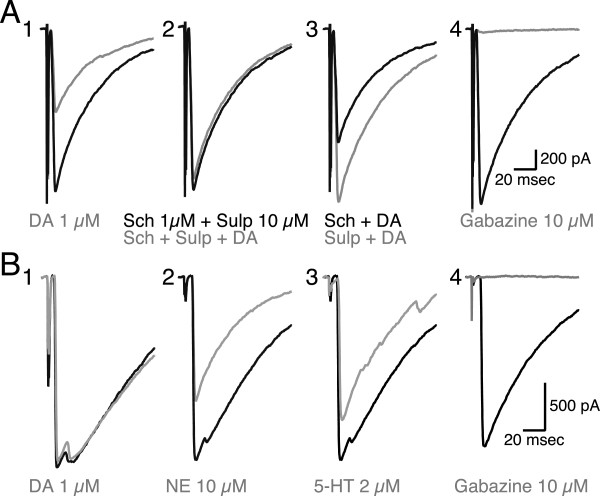
**Neurotransmitter modulation of autaptic response**. **A**. Here, a high-chloride intracellular solution was used so that autaptic responses were inward and larger. (A1) DA (*gray trace*) inhibited the autaptic response, here to 52% of control. (A2) Application of the D1 antagonist SCH23390 (SCH) together with Sulpiride (Sulp) had no impact on the response (*black trace*), but blocked DA action (*gray trace*). (A3) In this experiment, the DA inhibition was D2-mediated, as Sulpiride blocked DA action completely (*gray trace*), while SCH23390 had only a modest effect. (A4) At the end of the experiment, gabazine (gray trace) completely blocked the autaptic response. **B**. In another cell, (B1) the autaptic connection did not show DA modulation. However, (B2) Norepinephrine (NE; 68% inhibition) and (B3) Serotonin (5-HT; 78% inhibition) inhibited the autaptic response, arguing that DA does not act through other monoamine receptors. (B4) At the end of the experiment, the autaptic response was completely blocked by Gabazine.

DA modulated MSN-to-MSN connections, both autaptic and synaptic responses (Figure 1, Figure 3A). We measured DA modulation as the fraction of the preceding control response, to normalize for the decrease in IPSC amplitude over the course of experiments. However, even when the response had plateaued at a diminished level, DA modulation persisted. DA inhibited autaptic responses in the majority of experiments; in the others, it had no effect or facilitated responses (Table 1). The D1 agonist SKF38393 and the D2 agonist Quinpirole both showed a similar picture, with inhibition predominating. D1 activation produced slightly more facilitation. While the agonists mimicked DA action, they generally did not produce as robust modulation, even when applied together at equimolar concentrations to DA.

Tests for pharmacological specificity were confounded by the fact that the antagonists at higher concentrations inhibited autaptic responses. To obtain larger and more stable responses for these experiments, we used a high-chloride intracellular solution, which flipped the IPSCs, but did not appreciably alter the DA modulation. We found that SCH23390 at 50 μM produced a complete blockade (n = 3), probably due to a direct inhibition of Ca^2+ ^currents [40]. SCH23390 at 0.1 to 1.0 μM had a slight (<10%) inhibitory effect on autaptic responses, but completely blocked DA actions (n = 7). Similarly, the D2 antagonist Sulpiride inhibited (but did not block) autaptic responses at 50 μM; at 10 μM it had only a modest or no effect on autaptic responses (n = 6). Applied together (Figure 3A), however, these concentrations of SCH23390 and Sulpiride completely blocked DA modulation of autaptic responses (n = 6 of 6), arguing that the modulatory effects were mediated by DA receptors. DA in the presence of the D1 receptor antagonist SCH23390 had a similar incidence and efficacy as Quinpirole (n = 10); similarly, DA in the presence of the D2 antagonist Sulpiride (1 μM) resembled SKF38393 (n = 10). Overall, about 85% of cells that responded to DA showed both D1- and D2-mediated modulation, consistent with extensive colocalization of DA receptors on MSN presynaptic varicosities, or at least arguing that D1- and D2-like receptors are both present on the varicosities of single neurons (see below).

We showed previously that the GABA_B _agonist Baclofen (100 μM) produced robust presynaptic inhibition, blocking autaptic responses [38]. Autaptic responses were inhibited by the other monoamines norepinephrine (10 μM) or serotonin (2 μM). These modulatory actions were apparently independent of DA receptors, as there were instances (n = 2) where the autaptic response was sensitive to the other transmitters but insensitive to DA (Figure 3B). So, a single synaptic connection may be subject to modulation via a multiplicity of presynaptic receptors – both autoreceptors and heteroreceptors.

### Modulation is presynaptic

#### Paired-pulse electrophysiology

**Figure 4:**
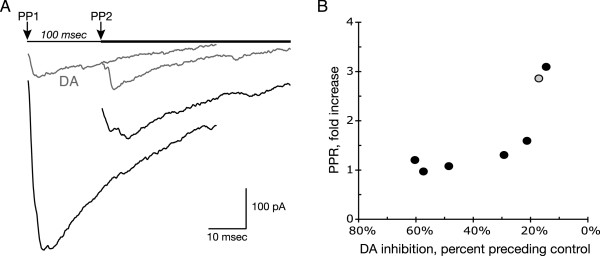
**Paired-pulse facilitation**. **A**. Paired pulses (PP1 and PP2) were delivered at a 100 msec interval in the absence (saline, *black traces*) and the presence of DA, 1 μM (*gray traces*). In this experiment, DA produced a dramatic inhibition to 17% of control, which was accompanied by about a 3-fold increase in the paired-pulse ratio (PPR), from 0.47 to 1.34. Traces shown are the averages of 5 traces. **B**. Plotting PPR *vs*. DA inhibition (expressed as the percent of the preceding control IPSC) for all paired-pulse experiments (n = 8), showed that PPR increased with DA modulation, indicative of presynaptic action. The gray-filled circle, corresponds to the experiment shown in panel A.

To examine the locus of modulation, we did paired-pulse experiments (Figure 4); an increase in paired pulse ratio (PPR) argues for presynaptic action [41,42]. In the dramatic experiment shown (Figure 4A), DA reduced the size of the IPSC to 17% of control and converted paired-pulse depression to facilitation, producing a 2.9 fold increase in the PPR. Overall, DA produced a 1.3 ± 0.2 fold increase in the PPR (n = 7). The magnitude of the increase correlated with the magnitude of DA modulation (Figure 4B).

#### FM1-43 destaining

**Figure 5:**
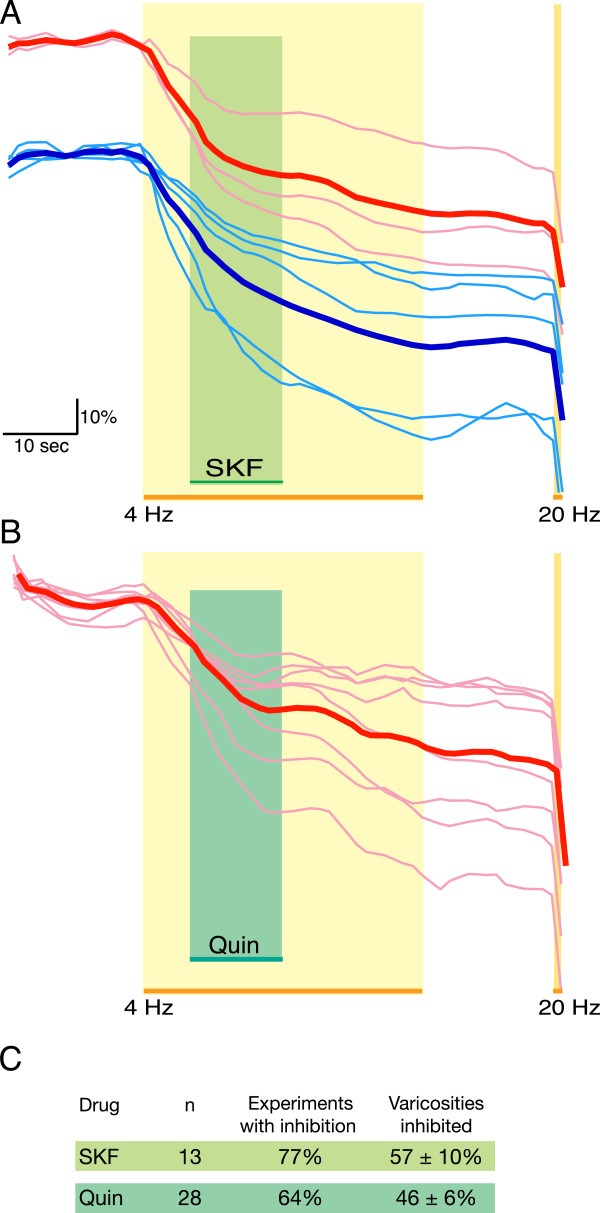
**Imaging presynaptic modulation by FM1-43 destaining**. Synaptic vesicles were loaded (stained) with FM1-43 by field stimulation, and then imaged every 1.25 sec. After a control period (15 images), showing that there was limited bleaching, field stimulation was applied at 4 Hz; destaining was monitored at 8varicosities (thin traces). **A**. During this stimulation, the D1 agonist SKF38393 (SKF,10 μM) was perfused (10 images). For 5 varicosities (*blue traces*), there was no inflection in the destaining curve, while in the others (*red traces*), the destaining was arrested, creating an inflection. The two thick traces are averages of the sets of effect and no-effect traces. The effect traces were offset upwards for the sake of the illustration. Once the SKF38393 was washed off, and the stimulation continued, destaining continued or resumed. After an interval of no stimulation, the remaining FM1-43 was unloaded with a 20 Hz tetanus. A total of 70 images were acquired. **B**. In another culture, the D2 agonist Quinpirole (Quin, 1 μM) was applied. In this experiment all 8 varicosities showed inhibition (red traces). D2 effects typically had a greater latency; note that the plateau started roughly when the drug was washed off (presumably coincidental). **C**. Overall, the majority of experiments showed either D1 or D2 inhibition, and in those experiments about half of varicosities showed inhibition (expressed as mean ± s.e.m.). Facilitation, as would be reflected in an increase in the destaining rate, was not seen. In each of the 13 D1 agonist experiments, a minimum of 2 varicosities showed inhibition; in the 18 D2 agonist experiments, 3 experiments had only one varicosity that showed inhibition. In one D1 agonist experiment and in one D2 agonist experiment every varicosity showed inhibition.

To show presynaptic modulation directly, we used the method of FM1-43 destaining [viz. [43,44]]. FM1-43 (10 μM) was loaded into vesicles by field stimulation (20 Hz; 30 sec), and then free FM1-43 was washed away. After initial images were acquired to establish the baseline, vesicles were unloaded with 4 Hz stimulation, during which drugs were applied by local perfusion. The intensity of staining at several synaptic sites was measured in CCD images acquired every 1.25 sec (Figure 5). As would be expected from its ability to completely inhibit GABAergic IPSCs [38], the GABA_B _agonist Baclofen (50 μM) completely arrested destaining (n = 4 of 4 experiments; data not shown). The D1 agonist SKF38393 (1 μM) or the D2 agonist Quinpirole (1 μM) each slowed or arrested destaining at about half of synapses in every experiment. The majority of experiments showed inhibition and in those experiments about half of the varicosities imaged showed inhibition (Figure 5C). We never saw an increased rate of destaining with SKF38393, as might be anticipated from the electrophysiological data. Since DA agonists produced at most a reduction to 50% inhibition of control GABAergic IPSCs (see previous section), while some varicosities showed arrest of FM1-43 destaining (0% of control), this suggests that a subset of MSN varicosities express DA receptors that may shut down release. As a control, we examined destaining in ventral tegmental area cultures, which lack D1 receptors, and confirmed that SKF38393 was ineffectual (n = 3 of 3; data not shown). So, both D1- and D2-like receptors appear to be present presynaptically and are thus positioned to modulate MSN synapses.

#### DA receptor visualization by immunostaining

**Figure 6:**
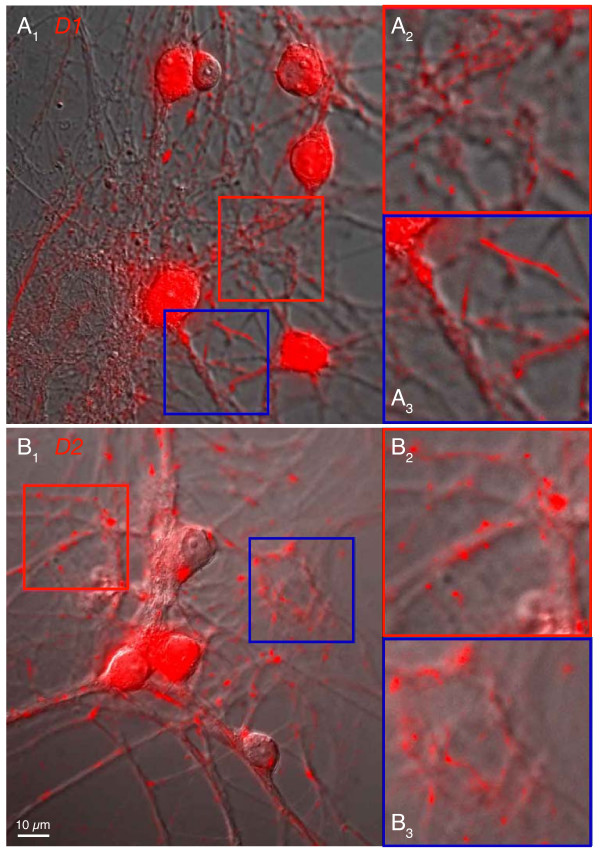
**DA receptor immunostaining shows a presynaptic pattern**. **A. D1 immunostaining**. (A1) A field in a nAcc culture is shown with fluorescence superimposed on a differential interference contrast (DIC) image. Here five of six cells were D1 immunoreactive. Note that the cell body staining did not extend reliably out onto the dendrites. Rather, punctate or linear staining was seen in the neuropil, consistent with staining of axons and presynaptic varicosities. Regions of interest outlined in red and blue are shown at 2× magnification on the right side. (A2) D1 fluorescence revealed strings of varicosities studding a thin process. (A3) In another region of the neuropil, continuous staining of putative-axonal processes was seen. **B. D2 immunostaining**. (B1) A field in a different nAcc culture containing four neurons, two of which were D2 immunoreactive, is shown. Again, note that the cell body staining does not extend continuously out onto the dendritic processes. Rather, punctate and linear staining of putative axonal processes is evident in the neuropil. (B2) Several varicosities without clear intervening axonal staining are seen studding unstained dendrites. A stretch of labeled axon is seen on the lower, right corner of the field. (B3) In another region, there were stained varicosities studding axonal processes. Note the dearth of postsynaptic dendritic labeling.

We showed previously by fluoroprobe labeling that D1 and D2 receptors are present on FM1-43-labeled presynaptic varicosities [29]. To gain higher resolution images of the distribution of DA receptors, we immunostained nAcc cultures with either D1-or D2-selective antisera (Figure 6). While the fluoroprobes revealed variations in postsynaptic membrane labeling, immunostaining showed patches of somatodendritic DA receptors and what appeared to be presynaptic labeling. Contrary to the fluoroprobe data where intravaricose axons were apparently unlabeled, immunostaining revealed not only varicosities but in places wisps of receptor labeling on connecting axonal segments, which by their thin, constant diameter confirmed axonal localization. Results shown were obtained with antipeptide antisera to rat D1a receptor [rabbit polyclonal to peptide 314, 3rd intracellular loop, Ref. [45]] and rat D2 receptor [rabbit polyclonal to peptide 53, extracellular amino terminus, Ref. [46]]. Similar results were obtained with antisera to human D1 receptor [clone 1-1-F11 s.E6 directed to the C-terminus, Ref. [47]] and the rat D2 receptor [extracellular amino terminus, Ref. [48]].

### DA differentially modulates two-component nAcc autaptic connections

**Figure 7:**
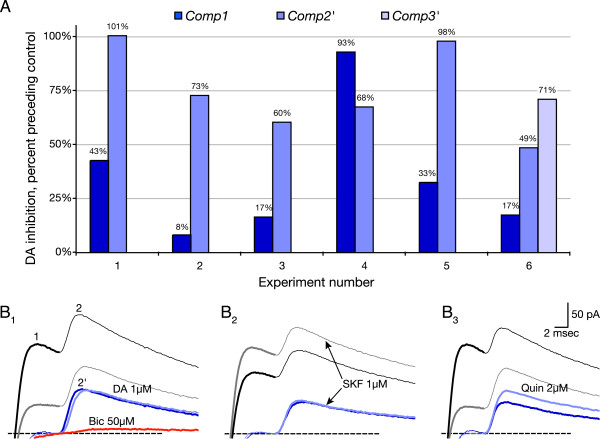
**Dopamine modulation of multicomponent responses**. **A**. In each of six experiments with multiple components, DA inhibited the components differentially. In experiment number 6, there were three components and each was differentially modulated. Component 1 (Comp1) was measured directly; components 2' and 3' were measured from the calculated responses after exponential extrapolation of the preceding component (or in experiment 6, the preceding two components). **B**. Differential DA modulation is illustrated in traces from experiment 5, in which we were successful in testing the effects of both D1 and D2 agonists. (B1) The control response had two components (1 and 2). Comp1 was extended by exponential curve fitting and then subtracted from the control trace to isolate Comp2' (*blue traces*). The sections of the traces measured (for Comp1 and Comp2') are shown thickened. DA (*gray thick trace*) inhibited Comp1 to 33% of control, while it had no effect on 2' (*light blue thick trace*). Bicuculline (Bic) blocked both components, nearly completely. (B2) SKF38393 (SKF) facilitated Comp1 (*gray thick trace*), but had no effect on Comp2' (*light blue thick trace*). (B3) Quinpirole (Quin), inhibited Comp1 (*gray thick trace*) and facilitated Comp2' (*light blue thick trace*). The dashed line indicates the zero current baseline.

In a few experiments (n = 6), stimulated action currents reliably evoked an initial autaptic response, which we called Component1 (Comp1), and after a fixed latency a second, smaller IPSC (Comp2), riding on top of the initial response (Figure 7); in one of these experiments, there was another, still further delayed, small IPSC (Comp3). All the components were GABA_A _mediated. The fixed delay was most likely due to conduction time in a recurrent axon branch that traveled away from the cell for some distance and then returned to make autaptic contacts. It could be argued that Comp2 was due to activation of a neighboring cell via depolarizing GABA action [49] or electrical coupling [17]; however, the latency was too short to accommodate an intervening synaptic connection, and did not show the variation that would be introduced by the firing of an intervening cell. Moreover, electrical coupling amongst MSNs tends to be too weak to drive postsynaptic cells to fire [17] under normal conditions [50]. Another explanation might be multivesicular release [51], but the latency of Comp2 was too long for this to be the mechanism.

DA differentially modulated the different autaptic components (Figure 7A). In the majority of the experiments DA modulation of Comp1 was the largest, but in no experiment was the modulation or the multiple components the same. The effects of selective agonists were tested successfully in experiment number 5 (Figure 7B). Exponential extrapolation of the decay of Comp1 and subtraction to isolate Comp2 (labeled 2') revealed that DA markedly attenuated Comp1, with little effect on Comp2' (Figure 7B1). In contrast, the D1 agonist SKF38393 facilitated Comp1 with no effect on Comp2' (Figure 7B2). The D2 agonist Quinpirole significantly attenuated Comp1 while modestly facilitating Comp2' (Figure 7B3). Since the two components must be regarded as being part of a single synaptic connection – just separated temporally by the conduction time due to an intervening length of axon – DA is apparently able to modulate release sites making up a single synaptic connection differentially.

In experiment 5, in which there was the greatest temporal separation of the components, we were able to compare the decay of the control responses and their rise time. As plotted in Figure 2B, Comp1 decayed over about 10 min to a relative plateau at about 25% of the initial value, presumably due to washout; in contrast, Comp2 was stable throughout most of the experiment (just over an hour), presumably because the intervening axonal segment retarded washout. The rise time of Comp1, 1.07 msec, was slightly faster than that of Comp2', 1.47 msec. The smaller amplitude and slower rise time of Comp2 would be expected if it was mediated by synaptic varicosities that impinged on more distal dendrites than those that mediated Comp1.

## Discussion

In postnatal nAcc cultures, we examined MSN-to-MSN synaptic connections and their modulation by DA. We focused on autaptic responses because they provided access to single, identified synaptic connections. Autaptic responses were subject to presynaptic DA modulation, with D2-like receptors predominantly mediating inhibition, and D1-like receptors more often mediating facilitation. The majority of connections showed both D1 and D2 modulation, consistent with significant receptor co-expression. In some cells, the autaptic responses had two or three components that were differentially modulated by DA, consistent with differential trafficking of DA receptors to different presynaptic varicosities of the same cell.

### Medium-spiny neuron connections *in vitro*

In postnatal culture, most MSN neurons show a single autaptic GABA_A _mediated, Ca^2+^-dependent after-hyperpolarization [38]. Some connections had a delayed component, with fixed latency, that we would argue was a second autaptic connection made after the MSN axon had traveled for some distance, introducing a conduction delay. MSN autapses have not been reported in the intact circuitry of the brain, and so they presumably arise in culture because of the artificial conditions, which include the immaturity of the cells, the lack of afferentation, the lack of normal projection targets, and the two-dimensionality of the cultures, any or all of which presumably contribute to the propensity of the cells to form autaptic connections. That being stated, MSN autapses do provide a powerful window on the synaptic capabilities of the cells, and serve to frame hypotheses that may be tested in the intact circuitry of the striatal complex, which we have now done [52].

Stimulating adjacent MSNs evoked a synaptic response of larger amplitude and faster rise time that was resistant to washout. Synaptic connections might impinge on more proximal somatodendritic membranes, consistent with recent observations suggesting that different populations of GABAergic inputs can be differentially distributed over the dendritic arbor [53]. The stability of the synaptic response confirmed that postsynaptic GABA_A _receptor sensitivity was maintained with the standard intracellular solution we used [54]. The single autaptic response, or the first component of the multicomponent autaptic response, showed a progressive decrease in amplitude over the course of about 15 min, which would be expected if the release sites mediating the response were close to the recording electrode in the cell soma and so subject to washout. The second component of the multicomponent autaptic response was typically smaller in amplitude and slower in rise time arguing that the recurrent axon made a more distal and thus weaker autaptic connection. Consistent with this, the second component was resistant to washout, presumably due to the functional diffusion limitation of the more distal axon. Interestingly, while the single autaptic component decreased to a fraction of its initial amplitude, the magnitude of DA modulation did not decrease.

The argument could be made that the single autaptic response – or the first component of the multicomponent response – was mediated by an autoreceptor current mechanism, as described by Pouzat and Marty in cerebellar interneurons [55]. Indeed, we observed strikingly similar washout kinetics. However, washout of key elements of the presynaptic machinery does not distinguish between an autoreceptor current (where the presynaptic varicosity is also the postsynaptic element) and an autaptic (axodendritic) response, but amplitude does. A comparison of the flipped IPSC elicited with similar high chloride-intracellular solutions used revealed that the autaptic responses we recorded were an order of magnitude larger. Given the two-dimensional neuropil of the cultures, which should limit the numbers of proximal presynaptic varicosities, we should have obtained the opposite result. A further comparison with their data revealed that second autaptic component they measured, which they deemed to be axodendritic, was in fact larger, rather than smaller, as we observed. The lack of evidence for GABA_A _receptors at presynaptic sites, either in the intact striatum [56] or in nAcc culture [29], argues further against the autoreceptor current mechanism. In our FM1-43 experiments, field stimulation that activated most synapses in the culture did not evoke sufficient GABA release to activate presynaptic GABA_B _receptors and arrest destaining. So there would not have been sufficient GABA release to activate presynaptic GABA_A _receptors, even if they were present, arguing further against the autoreceptor current mechanism. Therefore, the autaptic responses reported here, and in our previous study [38], are mediated by *bone fide *axodendritic autaptic connections.

### Dopamine modulation

DA was the most effective DA agonist, producing on average about a 50% inhibition of autaptic responses. In rare experiments, DA produced near complete inhibition. However, DA was not as robust as the GABA_B _agonist Baclofen, which consistently shut down the autapses [38]. DA and a combination of D1 and D2 agonists produced a similar incidence of modulation, but equimolar DA concentrations produced more robust inhibition (but not facilitation). Receptor family-selective modulation with DA and a single antagonist was also more robust that the corresponding single agonist. This was not due to DA acting via other monoaminergic receptors [viz. [57]], as there were clear instances where norepinephrine and serotonin modulated MSN synaptic connections that showed modest DA modulation. Moreover, the magnitude of the monoamine inhibition was no greater than the magnitude of DA inhibition, so it would be unlikely that DA, a poor serotonin or noradrenergic agonist, would have that much effect via the heterologous receptors. More likely, DA and the selective agonists activated different DA receptor confirmations with differing potencies, as has been described for both D1 and D2 receptors [58-61].

Most MSN connections showed both D1 and D2 modulation, consistent with near complete overlap in the expression of D1- and D2-like receptors on individual MSNs. Reports of a lesser incidence of colocalization (summarized in the Background) may be ascribed in part to detection issues with the different methodologies employed. It may be argued that physiological data, reflecting the actual function of the receptors, provide the most sensitive validation [31,62]. The apparent contradictions in the results could also be explained by differential trafficking of DA receptors (see below).

### Presynaptic DA modulation

Slice studies indicate that most DA modulation of MSNs arises through modulation of synaptic inputs onto the cells with only a minor postsynaptic contribution [63]. DA depresses GABA_A _responses via postsynaptic action, but the magnitude was on the order of 20% [64], whereas we observed a modulation on the order of 50%, arguing for an additional presynaptic action. Guzman *et al*. [9] provided the first evidence for presynaptic DA modulation in the striatal slice. Our paired-pulse experiments provide evidence for presynaptic modulation of MSN-to-MSN connections in culture. Direct imaging of DA modulation via inhibition of FM1-43 destaining showed that both D1 and D2 agonists arrested destaining. Although we cannot be certain that the varicosities we imaged all belonged to MSNs, the sheer numbers argue that we were. Moreover, MSNs show presynaptic modulation of their projection synapses [25,28], so the neurons are clearly capable of mounting presynaptic DA receptors.

Presynaptic actions of DA in the striatal complex have been called into question by the dearth of morphological evidence for presynaptic DA receptors [47,65-70]. However, there is extensive physiological evidence for presynaptic DA receptors on three classes of synapses in the striatal complex. Presynaptic D2 autoreceptors on DA neuron terminals inhibit DA release [71], D2 heteroreceptors on corticostriatal terminals inhibit glutamatergic inputs [72], and presynaptic D1 and D2 heteroreceptors on MSN terminals modulate GABAergic IPSCs [present results, and Refs. [9,73]]. DA receptors can be visualized using fluoroprobe antagonists on FM1-43 labeled presynaptic varicosities of both nAcc and striatal MSNs in postnatal culture [29]. When exogenous DA receptors tagged with yellow fluorescent protein (EYFP) are chemically transfected into nAcc cultures, they take up presynaptic positions [74]. Antipeptide antisera directed against D1 and D2 receptor epitopes mainly visualize receptors on axonal segments and presynaptic varicosities, cell bodies, but not dendrites. Possibly, presynaptic receptors are more easily solubilized [viz. Ref. [75]] with the usual concentrations of detergent used in preparing tissue for immunostaining, and so could account for an artifactual underestimate of the incidence of presynaptic DA receptors. Indeed, we have found that eliminating detergent from the immunostaining protocol dramatically enhanced presynaptic immnolabeling. In culture, the use of detergent is unnecessary presumably because the fixation process adequately permeabilizes cells. Consistent with this supposition, we obtained similar staining patterns using antipeptide antisera directed to extracellular and to intracellular epitopes (in the absence of detergent).

### Presynaptic modulation is heterogeneous

As suggested by Surmeier *et al*. [19], much of the controversy regarding the distribution of DA receptors on MSNs in the striatal complex would be resolved if MSNs traffic DA receptors to their presynaptic varicosities differentially. Otherwise it is difficult to integrate anatomical studies indicating that MSNs project sequentially rather than in parallel to their major target areas [32,33], with physiological studies showing D2 inhibition of MSN projections to pallidal neurons [28] and D1 facilitation on projections to the ventral midbrain [25], and data showing extensive co-expression of D1 and D2-like receptors in MSNs [29,31,62].

To show differential trafficking directly requires studies at the level of single cells. We have fortuitously found that some MSNs in postnatal culture show multicomponent autaptic responses. Since both responses share the same presynaptic and postsynaptic elements, they should be considered to be components of the same synaptic connection. And, given that they are connections between MSNs, they then represent the majority of intrinsic synapses in the striatal complex. We cannot rule out the possibility that the autaptic connections arise aberrantly in the absence of their projection targets, given the lack of documented autaptic connections in the intact circuitry. However, this does not detract from the fact that we have access to two separate populations of presynaptic varicosities that together make up a single MSN synaptic connection.

Given this fortuitous situation, we were able, in the experiments with two-component autaptic responses, to test the differential effect of DA agonists. If DA receptors were distributed homogeneously on all the presynaptic receptors of a given MSN, we would expect that both components of the autaptic response should show the same modulation. If the magnitude of the modulation differed, one could argue that the distribution was relatively homogenous, with variation in the level of expression at different varicosities. However, we found that D1 or D2 modulation might be lacking on one connection or have opposite effects, consistent with true differential trafficking of the receptors. The discrepancy between our FM1-43 experiments, which showed that varicosities were inhibited on an all-or-none basis, and the electrophysiology, which showed about a 50% inhibition, argues that a mix of unaffected and strongly modulated presynaptic varicosities accounts for the modulation we observed electrophysiologically. The immunocytochemical results that showed D1 or D2 receptors on only a subset of MSN varicosities argue against differential coupling to postreceptor mechanisms and for differential trafficking. In recent nAcc slice studies, we have shown that DA modulates Ca^2+ ^influx into the varicosities of single MSNs heterogeneously, providing further evidence for differential trafficking [52].

## Conclusion

Our present culture results, building on several lines of evidence and supported by recent slice studies, make the strong argument that individual MSNs differentially traffic DA receptors to their presynaptic varicosities. Definitive support for this argument will require imaging the distribution of DA receptors in single MSNs directly. We cannot exclude the possibility that the presence of aberrant projection synapses in the intrinsic population in our cultures accounts for the differential distribution of DA receptors we have observed. Gaining insight into the distribution of DA receptors on the local synapses of individual MSNs holds considerable import. DA is arguably the major modulator of information processing in the area. If DA receptors are differentially trafficked to MSN intrinsic synapses, and moreover if trafficking is plastic, then the distribution of presynaptic receptors would play a crucial role in the dynamic regulation of information processing in the striatal complex.

## Methods

### Pharmacologic agents

Dopamine, DA agonists and antagonists, as well as Gabazine (SR-95531; 2-(3-Carboxypropyl)-3-amino-6-(4 methoxyphenyl)pyridazinium bromide) were obtained from Sigma-Aldrich. We used Gabazine to avoid the confound that bicuculline salts block S-K^+ ^channels via a tetraethylammonium (quaternary ammonium group) like effect [76]. CNQX and CGP35348 were from Tocris-Cookson. FM1-43 was from Molecular Probes/InVitrogen. DA and all drugs used were made up in 1000× stock solutions, stored frozen at -80°C in aliquots, and thawed immediately before use. In initial experiments, ascorbic acid was included in the DA stock solution (at 10× the DA concentration) to prevent DA oxidation; however, this had no appreciable benefit, so the DA application experiments with and without ascorbic acid were pooled.

### Postnatal neuronal culture

We cultured neurons from the nucleus accumbens (nAcc) of postnatal (P2) Sprague-Dawley rat pups (Hilltop Lab Animals), with only minor modifications from our previously published methods [29,38]. Animal procedures were approved by the Institutional Animal Care and Use Committees of Columbia University and the New York State Psychiatric Institute, and were in compliance with the USPHS *Guide for the Care and Use of Laboratory Animals*. Pups were anesthetized with ketamine/xylazine, then chilled in ice chips, and decapitated. Brains were removed into chilled phosphate-buffered saline. An astrocyte feeder layer was prepared from the dorsal cortex two weeks in advance of making neuron cultures. For this, enzymatically-dissociated cortical cells were plated in the ~100 μL wells of glass-bottom dishes (MatTek) that had been coated with laminin; after 1 hour, wells were vigorously washed with cold medium leaving only strongly adherent astrocytes; once these grew to near confluence, further division was halted with fluorodeoxyuridine.

To obtain nAcc neurons, we cut the front half of the brain sagitally along the midline and made a 2 mm thick horizontal slice centered on the anterior commissure; further cuts were made using the ventral tip of the lateral ventricle as the medial cut to isolate the nAcc in a 2 × 2 × 2 mm cube. This was cut further into 1 × 1 × 1 mm segments and incubated in Papain (Worthington) at 32°C with gentle agitation and continuous carbogenation. After 90 min, the Papain was quenched with medium containing 10% calf serum and the tissue cubes dissociated to single cells by three rounds of gentle trituration in the presence of DNAase. Neurons were resuspended in B-27 supplemented NeurobasalA medium (Invitrogen), with 1% heat-inactivated supplemented-defined calf serum (Hyclone), added to assure glial longevity, and plated onto the pre-established monolayers of cortical astrocytes (in the microwells). Cultures were maintained in a total volume of 2.5 ml (which filled the whole dish), and never fed. To prevent microglial proliferation, fluorodeoxyuridine was added again after 7 days *in vitro *(DIV). Experiments were conducted on cultures between 10 and 21DIV, a period during which numbers of synapses are relatively stable [37].

### Electrophysiology

Recordings were done at room temperature (~22°C) on the stage of an inverted microscope (Zeiss IM35 or Axiovert35M). The culture medium was replaced with oxygenated extracellular solution containing 135 mM NaCl, 3 mM KCl, 2 mM CaCl_2_, 2 mM MgCl_2_, 10 mM glucose and 10 mM N-(2-Hydroxyethyl)piperazine-N'-(2-ethanesulfonic acid) (HEPES), pH7.35 (with KOH). Patch pipets, 6-9MΩ, were pulled on a P-80/PC Flaming-Brown Micropipet Puller (Sutter), and filled with either a standard intracellular solution that contained 140 mM gluconic acid, 140 mM KOH, 0.1 mM CaCl_2_, 2 mM MgCl_2_, 1 mM ethylene glycol-bis(2-aminoethylether)-N,N,N',N'-tetraacetic acid (EGTA), 2 mM ATP, 0.1GTP and 10 mM HEPES, pH7.25, or a high-chloride solution – transforming IPSCs into larger EPSCs – that contained 130 mM KCl, 0.1 mM CaCl_2_, 4.6 mM MgCl_2_, 10 mM HEPES, 1 mM EGTA, 4 mM ATP and 0.4 mM GTP, pH7.25.

Experiments were controlled by a Power Macintosh (Apple Computer) using IgorPro (Wavemetrics) with PulseControl XOP's [77]. Neurons were voltage clamped to -60 mV (to resolve autaptic currents and to prevent repetitive firing) using an Axoclamp2A (in continuous single electrode voltage clamp mode) or an Axopatch 200 (Axon Instruments/Molecular Devices). This holding potential did not include a correction for the liquid junction potential of about -15 mV, so the actual holding potential was about -75 mV. Membrane currents were digitized (Instrutech ITC-16 interface) and recorded (PulseControl) to disk. Compensation for passive conductance and series resistance was done by adding a scaled average of four -5 mV pulses delivered after each epoch of data acquisition (PulseControl: Subtraction Pulses Global). Further analysis of the data was done in IgorPro or Axograph (Axon Instruments/Molecular Devices).

Drugs were applied by local perfusion via a solenoid-controlled, gravity-fed Y-tube system; multiple drug-application channels were available via a rotary fluid switch (Rainin). Following solenoid activation, onset of drug perfusion was rapid, but delayed by about 5 sec, which was required for reversal of flow through the drug application tube. When the solenoid was deactivated, drug was rapidly removed by reverse flow through the drug-application tube. Fast green was used in some experiments to verify proper performance of the local perfusion system; it had no apparent pharmacologic effects at the concentration used. Drug effects were deemed significant if they exceeded 5%. To assure that drug application was uniform over the recorded cell, all experiments began with the application of bicuculline or gabazine, and were not continued unless better than 95% GABA blockade was obtained; as possible, experiments were also concluded with a test of GABA blockade, and if the blockade was not 95% or better, the experiments were discarded.

Unclamped A-spikes were evoked with 1 msec step depolarizations of about 30 mV to elicit autaptic currents. Voltage clamp control was such that we were often able to reestablish a brief zero-current epoch before the autaptic response. Synaptic responses were evoked by 1 msec depolarizing pulses delivered to the adjacent neuropil or cell bodies via a loose patch electrode; in some cases, the recorded cell was backfired, as was evident by an evoked action current spike; these responses were deemed autaptic, as they differed in no appreciable way from responses evoked by somatic depolarizing pulses. In contrast, synaptic responses showed no preceding inward current spike and rose cleanly from the baseline with a sharp inflection.

In most experiments, cells were stimulated at 0.1 Hz. Latencies of synaptic responses were measured from the peak of the action current, or from the stimulus artifact if its peak merged with the action current, to the point of maximum inflection at the onset of the synaptic response. Rise times were measured as the time from 10 to 90% of the peak amplitude of the synaptic current (AxoGraph). Five control responses were considered, then drugs was applied by local perfusion. After about 30 sec, which were required for responses to stabilize, five drug responses were then averaged, and the drug washed off. Again, after about 60 sec, when responses had again stabilized, five responses were averaged for a post-drug control. Numerical data were expressed as mean ± s.e.m. Significance of differences was evaluated by t-test.

In two-component responses, the second component, Comp2' was isolated by exponential curve fitting and subtraction. The assumption was made that the late decay (40 – 100 msec) of Comp2 would be representative of the decay of the first component, Comp1; indeed, in single component responses, the decay was well fit by a single exponential (IgorPro),

y=y0+A∗e−t0/τ
 MathType@MTEF@5@5@+=feaafiart1ev1aaatCvAUfKttLearuWrP9MDH5MBPbIqV92AaeXatLxBI9gBaebbnrfifHhDYfgasaacH8akY=wiFfYdH8Gipec8Eeeu0xXdbba9frFj0=OqFfea0dXdd9vqai=hGuQ8kuc9pgc9s8qqaq=dirpe0xb9q8qiLsFr0=vr0=vr0dc8meaabaqaciaacaGaaeqabaqabeGadaaakeaacqWG5bqEcqGH9aqpcqWG5bqEdaWgaaWcbaGaeGimaadabeaakiabgUcaRiabdgeabjabgEHiQiabdwgaLnaaCaaaleqabaGaeyOeI0IaemiDaq3aaSbaaWqaaiabicdaWaqabaWccqGGVaWliiGacqWFepaDaaaaaa@3C5E@

This late exponential curve fit was scaled to the amplitude of the first component (*A *set to ratio of the amplitudes of Comp1/Comp2) and shifted temporally by the interval (Δ*t*) between the components (*t*_0 _set to *t*_0_-Δ*t*). This provided an extrapolation of the decay of Comp1 uncontaminated by Comp2, which was then subtracted from the raw trace to yield a *pure *second component, Comp2'. In the experiments considered, Comp2' decayed properly to the baseline.

### Immunocytochemistry

Cultures were processed to visualize DA receptors following standard immunocytochemistry protocols. Briefly, cultures were slow fixed with 4%formaldehyde (prepared from 8% paraformaldehyde, EM Sciences, and 2× phosphate buffer) + 0.3% glutaraldehyde (Sigma-Aldrich). Polyclonal antipeptide antisera to D1a and D2 receptors (Marjorie A. Ariano, Rosalind Franklin University of Medicine and Science; Chemicon) were applied at 1:1000 dilution, overnight at 4°C, and then visualized using fluorescent secondary antisera (Chemicon). Detergent was not used. Sequences of images were acquired (Zeiss Axiovert; Scanalytics IP-Lab), at 0.5 μm intervals (Ludl Z-motor) using a Sensys chilled-CCD camera (Photometrics/Roper Scientific), and digitally deconvolved to produce confocal images (VayTek Microtome). The sequence of images encompassing the varicosities of interest were Z-projected and overlaid on the corresponding Nomarski-DIC images (Fluorescence CV, IP-Lab extension).

### Activity-dependent FM1-43 destaining

Synapses were loaded with FM1-43 (10 μM) by field stimulation (20 V applied between platinum electrodes, placed 1 cm apart on either side of the microwell) with a train of 1 msec duration pulses delivered at 20 Hz for 30 sec. After 5 min to allow for endocytosis, the FM1-43 was removed with three washes. The same imaging system as described above was used to image FM1-43 destaining, with the addition of a MacLab/8 (Analog Digital Instruments) for automation. The MacLab was programmed to deliver timed TTL pulses to trigger the camera, fluorescence shutter, stimulator, and the drug-application solenoid valve; pulse sequences were recorded (MacLab Chart software) to produce a graphical log. Images were acquired every 1.25 sec (200 msec exposure; camera gain on high), and after a control period, destaining was initiated by slow stimulation at 4 Hz. During this stimulation, drugs were applied by local perfusion for a discrete time interval; presynaptic inhibition would be expected to slow or arrest destaining, while facilitation would be expected to accelerate destaining. Stimulation was continued after drug application to confirm that destaining resumed. Stimulation was then stopped, and after images were acquired to establish the new baseline, a second tetanus (20 Hz for 20 sec) was delivered to unload remaining FM1-43 and confirm that activity-dependent destaining was not exhausted to rule out a floor effect. Experiments were only analyzed if they met the following criteria: a stable plateau prior to stimulation, destaining with the onset of stimulation, a stable post-stimulation baseline, and a final tetanus-induced destaining. In some experiments, to test whether stimulated GABA release might reduce the efficacy of stimulation for loading or destaining via presynaptic inhibition, the GABA_B _antagonist CGP35348 (500 μM) was included throughout; however, this made no appreciable difference, so experiments with and without CGP35348 were pooled in the data tabulation.

For analysis, 10 regions of interest (ROI's) 2 μm square were selected – nine ROI's were of individual varicosities that were easily resolved from adjacent varicosities, and one ROI of an area containing no varicosities for determination of background staining. The intensity of the background ROI was subtracted from the other intensity measurements at each time point. Intensities were normalized to the average intensity of the initial control frames. There were occasional fluctuations in the intensity of the mercury arc, which were reflected by simultaneous jumps in the intensity in all 10ROI's; these points were removed and the average of the two adjacent time points used instead. Finally, traces were smoothed using a fifth power Gaussian algorithm (IgorPro).

We used a Monte Carlo simulation to evaluate the significance of the destaining results. An ideal no-effect curve was generated and scaled noise added to reproduce the appearance of the actual data. We then scored four series of 250 simulations to determine how often this noise level produced an apparent effect. We applied the same criteria used for the actual data, namely a readily evident slowing of destaining shortly after the onset of drug application and a resumption of destaining after drug application was stopped. In the four simulations, we observed an effect in 0.9 ± 0.3% of the traces, making p < 0.01 that the results we observed arose by chance.

## Authors' contributions

DG, JMN, and JJS did the electrophysiology; IGF did the FM1-43 imaging experiments; MPJ did the immunocytochemistry and provided statistical input; SR conceived and directed the project, and wrote the manuscript. All authors read and approved the final manuscript.

## Figures and Tables

**Table 1:** DA modulation of autaptic connections. Data are organized by the receptor targeted. The Incidence data give the percent of connections that showed modulation greater than 5%; less that a 5% modulation is reported as Neither. The Magnitude data (expressed as the percentage of the preceding control response) give the average size of the inhibited or facilitated IPSC for those connections showing inhibition or facilitation, respectively. DA or the combination of the D1 agonist SKF38393 (SKF) and the D2 agonist quinpirole (Quin) targeted DA receptors as a class. SKF38393 or DA in the presences of Sulpiride (Sulp) targeted D1 receptors. Similarly, Quinpirole or DA in the presence of the D1 antagonist SCH23390 (SCH) targeted D2 receptors. The other monoamines norepinephrine (NE) and serotonin (5-HT) were also tested. n/a, not applicable.

			**Incidence, %**			***Magnitude, %***	
				
*Targeted Receptor*	***Drug***	***n***	Inhibition	Facilitation	Neither	*Inhibition*	*Facilitation*
*D1, D2*	DA	*29*	83	10	7	*49*	*117*
	SKF & Quin	*4*	75	0	25	*88*	*n/a*
*D1*	SKF	*12*	50	33	17	*84*	*136*
	DA & Sulp	*10*	60	20	20	*54*	*127*
*D2*	Quin	*14*	71	21	7	*66*	*109*
	DA & SCH	*10*	70	10	20	*59*	*110*
*Monoamine*	NE	*2*	100	0	0	*48*	*n/a*
	5-HT	*5*	80	0	20	*52*	*n/a*
